# Biomass productivity and productivity of fatty acids and amino acids of microalgae strains as key characteristics of suitability for biodiesel production

**DOI:** 10.1007/s10811-012-9795-3

**Published:** 2012-02-11

**Authors:** Niels Hempel, Ingolf Petrick, Frank Behrendt

**Affiliations:** 1 Faculty of Natural Sciences, Lausitz University of Applied Sciences, Großenhainer Straße 57, 01968 Senftenberg, Germany; 2 Department of Energy Engineering, Berlin Institute of Technology, Fasanenstraße 89, 10623 Berlin, Germany

**Keywords:** Amino acids, Biodiesel, *Chlorella*, Fatty acid productivity, Species selection

## Abstract

Microalgae are discussed as an alternative source for the production of biofuels. The lipid content compared to cultivation time of used species is the main reason for any choice of a special strain. This paper reviews more analytical data of 38 screened microalgae strains. After the cultivation period, total content of lipids was analysed. The extracted fatty acids were quantified as fatty acid methyl esters by GC analysis. The amino acids were analysed by HPLC. *Chlorella* sp., *Chlorella saccharophila*, *Chlorella minutissima* and *Chlorella vulgaris* were identified as species with the highest productivity of fatty acids relevant to transesterification reactions. The components were mainly linoleic acid, palmitic acid and oleic acid. To increase productivity of highly saturated fatty acids, cultivation parameters light intensity and temperature were varied. In this manner, the ideal conditions for biodiesel production were defined in this publication.

**Electronic supplementary material:**

The online version of this article (doi:10.1007/s10811-012-9795-3) contains supplementary material, which is available to authorized users.

## Introduction

For environmental and economic sustainability of the world, renewable and carbon-neutral biofuels are necessary (Patil et al. 2008). Compared to conventional energy sources, alternative possibilities have only a minor economic basis. Today, optimisation of production processes is the main reason for increasing research activities in this area. A multi-faceted approach for an energy generation that includes biofuels as well as nuclear, solar, hydrogen, wind and fossil fuels (from which carbon is sequestered) will provide the backbone for industries in any future world (Hoffart et al. 2002; Pacala and Socolow 2004; Patil 2007). Alternative routes will reduce greenhouse gases such as carbon dioxide (CO_2_), sulphur dioxide (SO_2_) and nitrogen oxides (NO_x_) and help to maintain our high living standards in a less negatively affected environment.

Besides economic reasons, consumption of vast swathes of farmland and native habitats with increasing food prices are a major criticism against biomass usage for large-scale production (Crutzen et al. 2007; Pacala and Socolow 2004; Righelato and Spracklen 2007), the usage of agricultural crops alone unable to satisfy the entire demand. Oil palm crops, with their high content of oil, used for 50% of US transport fuel, would need 24% of the total cropland area of the USA (Chisti 2007). The resulting ‘food versus fuel’ argument is already widely discussed. In contrast, serious studies postulate that biofuels are able to reach an amount of 30% of global demand in an environmentally responsible manner without affecting food production (Koonin 2006).

Biomass is principally able to be used for production of various energy storaging substrates such as biodiesel, bioethanol, biomethane and biohydrogen, with only biodiesel and bioethanol produced to industrial quantities at this time (Patil et al. 2008). First-generation biofuels have already been displaced by second-generation biofuels produced by non-food feedstocks. Lignocellulosic technologies and microalgae play an important role in this manner (Chisti 2007; Hankamer et al. 2007; Kruse et al. 2005; Schaub et al. 2007). A wide range of advantages shift the focus to microalgae biomass. Microalgae are miniature biochemical factories using similar photosynthetic processes in the same manner as higher plants for generation of energy and intermediates for metabolism. Photosynthetic efficiency of microalgae, however, is notably higher than in terrestrial plants (Pirt 1986; Shay 1993). Microalgae already reach up to 300 times more oil productivity for biodiesel production than traditional crops on an area basis (Chisti 2007; Schenk et al. 2008). Alongside these advantages, microalgae assimilate CO_2_ into their biomass resulting in a reduction of concentrations of greenhouse gasses (Brown and Zeiler 1993). Furthermore, microalgae can be harvested in continuous processes (1–10 days depending on culture volume and culture techniques) enabling a high efficiency of cultivation processes. In contrast, conventional crop plants usually can only be harvested once or twice a year. Growing of crops is only possible in vegetation periods dependent on climatic and geographic requirements. Cultivation of microalgae can potentially be carried out on marginal or non-arable land allowing the possibility of opening up new economic opportunities for arid, drought- or salinity-affected regions (Chisti 2007). Greater photosynthetic efficiency results in reduced fertiliser and nutrient inputs and less waste and pollution, with the usage of waste water instead of drinking water for algal cultivation also being a viable option (Hammouda et al. 1995; Mallick 2002; Muñoz and Guieysse 2006). Compared to conventional agricultural production, the evaporation loss of fresh water is reduced during algal cultivation in closed systems. In addition, water can be used in a cycling system, thus considerably increasing water balance. Cultivation systems for microalgae can consist of open ponds, bubble columns, flat-plate photobioreactors (PBRs) or tubular PBRs (Borowitzka 1997; Ugwu et al. 2008; Xu et al. 2009). For large-scale production, PBRs are the most promising systems because of low contamination risk, low space requirement, almost no CO_2_ loss, high adaptability to many species and high biomass concentration (Pulz 2001; Richmond 2000). Growing of microalgae strains in closed systems under defined conditions allows usage of biomass for other applications such as production of foods. The utilisation of amino acids of algae biomass alongside fatty acids increases the economic basis of the biodiesel production process. However, for user acceptance, microalgal biodiesel needs to comply with existing standards, such as ASTM Biodiesel Standard D 6751 (USA) or Standard EN 14214 (European Union) meaning a high cetane number is therefore required. The cetane number is directly related to good performance characteristics of biofuel, showing ignition quality in an engine (Van Gerpen 2009). Highly saturated fatty acids give an excellent cetane number and oxidative stability to biodiesel (Chinnasamy et al. 2010). The content of such kinds of fatty acids is dependent on species-specific enzyme configuration of microalgae strains, with cultivation conditions also having a possible influence (Sato et al. 1979).

The first step in developing such an algal process is the screening of various strains to choose the most suitable species (Pulz and Gross 2004) with a high productivity of fatty acids. Relevant properties for specific culture conditions, as well as the additional identification of high valuable byproducts, play an important role. In this publication, the screening and characterisation results of 38 microalgae strains are given for the identification of potent biodiesel producers.

## Materials and methods

Strains and culture media used in this work are listed in Table 1. The strains were obtained from UTEX Culture Collection of Algae, Austin; SAG Culture Collection of Algae, Göttingen; SAS Culture Collection of Algae, Senftenberg; MACC Algal Culture Collection, Mosonmagyaróvár; and IPPAS Culture Collection of Microalgae, Moscow. The following culture media were used:

**Table 1:** List of laboratory algal strains (screened strains and reference strain), cultivation media and culture collection

Medium^a^	Algal strain (strain numbers; culture collection)
T	*Chlorella fusca* strain 251 (UTEX, Austin)
T	*Chlorella minutissima* strains 444; 452; 494 (MACC, Mosonmagyaróvár)
T	*Chlorella saccharophila* strains 363; 477 (MACC, Mosonmagyaróvár)
T	*Chlorella* sp. strains 03; 04; 11; 17-1; 18 (SAS, Senftenberg)
T	*Chlorella* sp. strains 4; 313; 318; 391; 418; 459; 474; 550; 552; 572; 589; 732; 800 (MACC, Mosonmagyaróvár)
T	*Chlorella vulgaris* strains 125; 132^b^ (SAS, Senftenberg)
T	*Chlorella vulgaris* strains 211-1b; 211-11f; 211-11j (SAG, Göttingen)
T	*Chlorella vulgaris* strain C1 (IPPAS, Moscow)
T	*Chlorella zofingiensis* strain 133 (SAS, Senftenberg)
B	*Chlorococcum ellipsoideum* strain 33 (MACC, Mosonmagyaróvár)
B	*Cosmarium* sp. strain 25 (SAS, Senftenberg)
T	*Neochloris* sp. strain 421 (MACC, Mosonmagyaróvár)
B	*Pediastrum boryanum* strain 39 (SAS, Senftenberg)
B	*Phaeodactylum tricornutum* strain 1090 (SAG, Göttingen)
T	*Scenedesmus rubescens* strain 5.95 (SAG, Göttingen)
S	*Spirulina maxima* strain 20 (SAS, Senftenberg)
S	*Spirulina platensis* strain 85.79 (SAG, Göttingen)

^a^Key to media: *B* modified BG11, *S* spirulina, *T* modified Tamiya

^b^Reference strain

Modified BG11 medium contained (g L^−1^): NaNO_3_, 1.5; MgSO_4_·7H_2_O, 0.075; K_2_HPO_4_, 0.035; CaCl_2_·2H_2_O, 0.036; Na_2_CO_3_, 0.02; ferric ammonium citrate, 0.006; citric acid, 0.006; Na_2_EDTA, 0.001; and trace elements solution, 1 mL L^−1^. The trace elements solution contained (g L^−1^): H_3_BO_3_, 0.061; MnSO_4_·H_2_O, 0.169; ZnSO_4_·7H_2_O, 0.287; CuSO_4_·5H_2_O, 0.0025; and (NH_4_)_6_Mo_7_O_24_·4H_2_O, 0.0125.

Spirulina medium contained (g L^−1^): NaHCO_3_, 6.8; Na_2_CO_3_, 2.0; K_2_HPO_4_, 0.25; NaNO_3_, 1.25; K_2_SO_4_, 0.5; NaCl, 0.5; MgSO_4_·7H_2_O, 0.1; CaCl_2_·2H_2_O, 0.02; FeSO_4_·7H_2_O, 0.005; Na_2_EDTA, 0.04; cyanocobalamine, 0.000005; and trace elements solution, 5 mL L^−1^. The trace elements solution contained (g L^−1^): ZnSO_4_·7H_2_O, 0.001; MnSO_4_·4H_2_O, 0.002; H_3_BO_3_, 0.01; Co(NO_3_)_2_·6H_2_O, 0.001; Na_2_MoO_4_·2H_2_O, 0.001, CuSO_4_·5H_2_O, 0.000001; FeSO_4_·7H_2_O, 0.7; and Na_2_EDTA, 0.8.

Modified Tamiya medium contained (g L^−1^): KNO_3_, 2.5; KH_2_PO_4_, 0.625; MgSO_4_·7H_2_O, 1.25; FeSO_4_·7H_2_O, 0.0045; Na_2_EDTA, 0.0186; and trace elements solution, 0.5 mL L^−1^. The trace elements solution contained (g L^−1^): H_3_BO_3_, 2.86; MnCl_2_·4H_2_O, 1.81; ZnSO_4_·7H_2_O, 0.222; NH_4_VO_3_, 0.023; and MoO_3_, 0.018.

Each microalgal strain was precultured in Erlenmeyer flasks: temperature 25°C; shaken at 80 rpm. For growth assay, 0.2 L culture suspension with dry weight (dw) of 2.0 g L^−1^ was inoculated in 2 L glass cylinder photobioreactors containing 1.8 L liquid medium (Table 1). Sterile filtered air enriched with 2% (v/v) CO_2_ was continuously pumped through special nozzles and glass tubes into the bottom of a photobioreactor. The aeration was performed at 0.33 L min^−1^ for 24 h. Cultures were incubated at 25°C with continuous stirring. The photobioreactors were illuminated with eight horizontally fixed 40 W white lamps which were placed behind the photobioreactors. The light intensity was approximately that of 200 μmol photons m^−2^ s^−1^. Each strain was cultivated in duplicate. To exclude a nitrate or phosphate limitation, the contents were determined every 2 or 3 days by using special test kits (Machery-Nagel, Germany). For a good comparability of results, all screened microalgae strains were harvested at 3 g L^−1^ by centrifugation at 4,050 × *g* for 10 min and washed with distilled water. Cell pellets were freeze-dried and stored in polypropylene cups until further analysis was conducted.

### Biomass assay

Cell growth was monitored by gravimetric determination of algal biomass dry weight (dw) with each determination made in duplicate every 2 or 3 days. Aliquots of 10 mL algal suspension were centrifuged at 4,050 × *g* for 10 min, washed with distilled water to remove the salt, dried at 105°C for 24 h to a constant weight, cooled in a desiccator and weighed. The growth curves were determined from parallel cultures starting from the same inocula and were calculated from average algal biomass as function of time.

Volumetric biomass productivity *P*_Biomass_ was calculated by ${P_{\text{Biomass}}}\left( {{\text{g}} \, {{\text{L}}^{ - {1}}}{\text{da}}{{\text{y}}^{ - {1}}}} \right) = \left( {{X_2} - {X_1}} \right) \cdot {\left( {{t_2} - {t_1}} \right)^{ - {1}}}$ where *X*_1_ and *X*_2_ were the biomass dw concentrations (g L^−1^) on days *t*_1_ (startpoint of cultivation) and *t*_2_ (endpoint of cultivation), respectively, and was given as an average productivity.

### Analysis of lipid content and fatty acid profile

Lipid content of algae probes was determined gravimetrically. One gram of freeze-dried biomass was resuspended in 6 mL of chloroform/methanol (2:1, v/v) and transferred to a grinding beaker with 4 g steel balls. The algae cells were disrupted in the ball mill (Retsch, Germany) at full activity (1,800 min^−1^) for 25 min and afterwards centrifuged at 4,050 × *g* for 10 min. Following centrifugation, the supernatant was filtered and collected. The rotary evaporator was then used to separate the chloroform and methanol from the dissolved oil fraction, with the residual oil being dried in an oven until constant weight was reached.

The amount of fatty acids was estimated as the amount of FAMEs, which was determined by direct transesterification. The dried lipid extract was dissolved in 10 mL *n*-heptane, and 10 mL 0.5 N methanolic KOH was added with the resulting mixture shaken for 30 min. Aliquots of upper phase with FAMEs were analysed using standard gas chromatography with a capillary column (100 m, 0.25 mm, 0.2 μm; SP™-2560; Supelco, USA) and a flame ionisation detector (7820 A, Agilent Technologies, Germany). H_2_ at 35 mL min^−1^ was used as carrier gas. Temperature was programmed to increase from 50 to 65°C at 2°C min^−1^ then to 180°C at 30°C min^−1^ and thereafter ramped to 220°C at 15°C min^−1^. The injector and detector were kept at 220°C and 250°C, respectively. The amount of FAMEs was quantified to comparison to a standard solution containing 37 FAMEs (Supelco, USA).

### Determination of amino acid profile

Amino acids were quantified using HPLC System S433 (Sykam, Germany). Then 100 mg of freeze-dried biomass was resuspended in 5 mL 1 N HCl with 1% (w/v) phenol and was rinsed with nitrogen gas for 1 min. Solutions were dried at 110°C for 24 h and filtered. Probes were evaporated, dissolved in a sample solution buffer and injected on a cation separation column (4.6 × 150 mm, LCA K06/Na; Sykam, Germany) and were detected at 440 nm and at 570 nm. The analyses were run under the following conditions: analysis cycle time 60 min; flow rates 0.45 mL min^−1^ for buffer and 0.25 mL min^−1^ for ninhydrin. The amino acid content is given as the summed content of alanine, asparagine, aspartic acid, arginine, cysteine, glutamic acid, glutamine, glycine, histidine, isoleucine, leucine, lysine, methionine, phenylalanine, proline, serine, threonine, tyrosine and valine. Based on acidic hydrolysis, the content of asparagine as well as aspartic acid and the content of glutamine and glutamic acid are given as the summed content of both amino acids.

### Analytics and calculation of productivities

All determinations were made in duplicate and were given as average content or average productivity. To exclude drift of analytical data, in addition to internal controls, the reference strain *Chlorella vulgaris* 132 was analysed every 3 months.

Productivity of lipids, fatty acids and amino acids were calculated by

$$ {P_{{\text{lipids}},{\text{ fatty acids}},{\text{ amino acids}}}}\left( {{\text{mg }} \, {{\text{L}}^{ - {1}}}{\text{da}}{{\text{y}}^{ - {1}}}} \right) = {P_{\text{Biomass}}} \cdot {C_{\text{f}}} $$

where *P*_lipids, fatty acids, amino acids_ is productivity of lipids, fatty acids or amino acids; *P*_Biomass_ is productivity of biomass; and *C*_f_ is the final content of lipids, fatty acids or amino acids and were given as percent dry weight.

For identification of productivities influencing factors in this publication, various analytical parameters were correlated. For linear trendlines, added to the data series, coefficients of determination (*r* ^2^) were determined and the levels of mutual influences were estimated.

## Results

### Biomass productivity

To test the suitability of microalgae for biodiesel production, screening data were collected for 35 strains of the green algae (Chlorophyta), two strains of the blue-green algae (cyanobacteria) and one strain of diatoms (Bacillariophyta). For all strains, growth curves were determined. No lag phase was observed, possibly because exponentially growing cells of precultures were used as inocula. Because of stagnating growth, *Phaodactylum tricornutum*, *Spirulina maxima* and *Spirulina platensis* were harvested prematurely (end-dw ranged between 1.0 and 2.2 g L^−1^) before reaching the end concentration of 3 g L^−1^. The stationary growth phase was not reached in any case, indicating no limiting conditions. Because of linearity of all growth curves, the biomass productivity was calculated over the whole cultivation period.

**Fig. 1:**
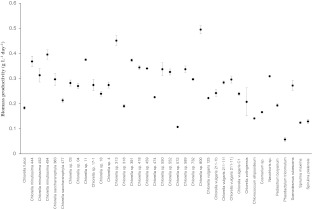
Average specific biomass productivity of 38 screened microalgae strains. For reference strain *Chlorella vulgaris* 132, the biomass productivity ranged between 0.38 and 0.435 g L^−1^ day^−1^ over the whole test period. *Error bars* show the minimum and the maximum recorded for each species

Figure 1 shows biomass productivity of all tested strains. *Chlorella* sp. 800, *Chlorella* sp. 313 and *Chlorella minutissima* 494 were identified as fast-growing strains with the highest biomass productivity. For *Chlorella* sp. 800, biomass productivity of 0.495 g L^−1^ day^−1^ was calculated, for *Chlorella* sp. 313 of 0.451 g L^−1^ day^−1^ and for *C. minutissima* 494 of 0.396 g L^−1^ day^−1^. Reference strain *C. vulgaris* 132 reached values of between 0.38 and 0.435 g L^−1^ day^−1^, indicating a good validity of growth data.

To increase biomass productivity of *Chlorella* sp. 800, the influence of light intensity and cultivation temperature was examined. At varied light intensity, the biomass productivity was 0.423 g  L^−1^ day^−1^ at 200 μmol photons m^−2^ s^−1^, 0.475 g L^−1^ day^−1^ at 300 μmol photons m^−2^ s^−1^, 0.62 g L^−1^ day^−1^ at 400 μmol photons m^−2^ s^−1^ and 0.707 g L^−1^ day^−1^ at 500 μmol photons m^−2^ s^−1^. A continuous increase of biomass productivity was postulated, and therefore a saturation effect of light was not detected. In further studies, a cultivation temperature of 25°C was identified as optimum. The biomass productivity was 0.538 g L^−1^ day^−1^ compared to 0.435 g L^−1^ day^−1^ at 35°C and 0.34 g L^−1^ day^−1^ at 15°C.

### Lipid productivity and productivity of fatty acids

**Fig. 2:**
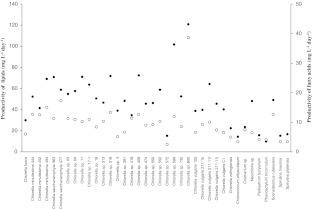
Specific lipid productivity (*filled circles*) and specific productivity of fatty acids (*open circles*) of 38 screened microalgae strains. For reference strain *Chlorella vulgaris* 132, the lipid productivity ranged between 80.5 and 88.5 mg L^−1^ day^−1^ over the whole test period and the productivity of fatty acids ranged between 12.6 and 15.6 mg L^−1^ day^−1^ over the whole test period

Strains with the highest lipid content were *Chlorella* sp. 589 (30.2% dw), *Chlorella saccharophila* 477 (27.6% dw) and *Chlorella* sp. 800 (24.4% dw), shown in Online Resource 1. For reference strain, analysed lipid content ranged between 18.5% and 23.3% dw. The resulting lipid productivity (Fig. 2) was recorded as follows: Based on both high biomass productivity and high lipid content, *Chlorella* sp. 800 was identified as the strain with the highest lipid productivity of 121 mg L^−1^ day^−1^. Strain *Chlorella* sp. 589 reached 102 mg L^−1^ day^−1^ and *Chlorella* sp. 459 72.5 mg L^−1^ day^−1^. For reference strain *C. vulgaris* 132, over the whole test period, lipid productivity between 80.5 and 88.5 mg L^−1^ day^−1^ was measured.

For estimation of content of fatty acids in screened microalgae strains, clear identifiable fatty acids were summarised (Online Resource 1). *C. saccharophila* 477 reached a content of 8.1% dw for fatty acids, *Chlorella* sp. 800 a content of 7.8% dw and *P. tricornutum* 7.2% dw. These strains were identified as species with the highest content of fatty acids of all screened strains. Despite 37 detectable fatty acids, only six fatty acids formed the main fraction: palmitic (C16:0), palmitoleic (C16:1), stearic (C18:0), oleic (C18:1ω9), linoleic (C18:2ω6) and α-linolenic (C18:3ω3). Summarised content of these fatty acids give more than 90% of all fatty acids. For most productive strains, these contents and productivities are given in Table 2. Differences were detected in contents of linoleic acid and α-linolenic acid. Strain *Chlorella* sp. 800 contained high amount of tri-unsaturated α-linolenic acid compared to di-unsaturated linoleic acid, indicating a high desaturase activity. In contrast, the remaining most productive strains had low comparable desaturase activity based on quantified fatty acids. Besides main fraction of fatty acids in six strains, divergent spectra of fatty acids were assumed. The reason for this phenomenon was founded in abnormal content of special fatty acids. High content of trans-configured linolelaidic acid (C18:2ω6t) was measured in *Chlorella* sp. 391, in *Cosmarium* sp. and in *Chlorococcum ellipsoideum*. For γ-linolenic acid (C18:3ω6), high content was detected in *C. ellipsoideum*, of *Cosmarium* sp. and of *S. platensis*. Extreme low content of linoleic acid (C18:2ω6) was the reason for the discrepancy in main spectrum of fatty acids in *Chlorella* sp. 391 and in *Chlorella zofingiensis*. In *P. tricornutum* biomass, a high amount of eicosapentaenoic acid (C20:5ω3) was detected.

**Table 2:** Content and productivity of fatty acids palmitic, palmitoleic, stearic, oleic, linoleic and α-linolenic of five microalgal strains

Fatty acid	Strains with highest productivity of fatty acids				
	*Chlorella* sp. 800	*Chlorella saccharophila* 477	*Chlorella minutissima* 494	*Chlorella* sp. 313	*Chlorella minutissima* 444
Palmitic (C16:0)					
Content (% dw)	1.86	1.56	1.18	1.08	1.00
Productivity (mg L^−1^ day^−1^)	9.21	3.33	4.67	4.87	3.68
Palmitoleic (C16:1)					
Content (% dw)	0.055	0.045	0.037	0.032	0.041
Productivity (mg L^−1^ day^−1^)	0.27	0.096	0.15	0.14	0.15
Stearic (C18:0)					
Content (% dw)	0.60	0.35	0.036	0.04	0.04
Productivity (mg L^−1^ day^−1^)	2.97	0.74	0.14	0.18	0.15
Oleic (C18:1ω9)					
Content (% dw)	2.59	1.86	0.18	0.16	0.14
Productivity (mg L^−1^ day^−1^)	12.80	3.96	0.71	0.72	0.52
Linoleic (C18:2ω6)					
Content (% dw)	1.97	4.04	2.05	1.41	1.95
Productivity (mg L^−1^ day^−1^)	9.80	8.61	8.09	6.36	7.17
α-Linolenic (C18:3ω3)					
Content (% dw)	0.67	0.19	0.25	0.19	0.23
Productivity (mg L^−1^ day^−1^)	3.31	0.40	0.99	0.86	0.85
Sum of fatty acids					
Content (% dw)	7.75	8.04	3.73	2.91	3.40
Productivity (mg L^−1^ day^−1^)	38.30	17.14	14.78	13.13	12.52

The determination of volumetric productivity of summarised fatty acids allows prediction of amount of synthesised fatty acids, both per day and per litre. Results for screening strains are given in Fig. 2. Strain *Chlorella* sp. 800 was identified as the top fatty acid producer. The productivity of fatty acids was 38.7 mg L^−1^ day^−1^, based on both fast-growing and high content of fatty acids. The next most productive strains were *C. saccharophila* 477 (17.3 mg L^−1^ day^−1^) and *C. minutissima* 494 (15.1 mg L^−1^ day^−1^). Reference strain also reached high fatty acid productivity, 12.6–15.6 mg L^−1^ day^−1^ over the whole test period.

For increasing productivity of fatty acids, cultivation temperature and light intensity were varied for top fatty acid producer strain *Chlorella* sp. 800. Cultivation at other temperatures than at room temperature resulted in a decreased fatty acid productivity: Compared to 27.4 mg L^−1^ day^−1^, value reached in these experiments for cultivation temperature of 25°C, at 15°C fatty acid productivity decreased by 54% and at 35°C by 64%. At increasing light intensity, an increase of fatty acid productivity was postulated: At 500 μmol photons m^−2^ s^−1^, the fatty acid productivity increased by 26% compared to cultivation at 200 μmol photons m^−2^ s^−1^.

### Variation of cultivation parameters and influence to quality of biodiesel

**Fig. 3:**
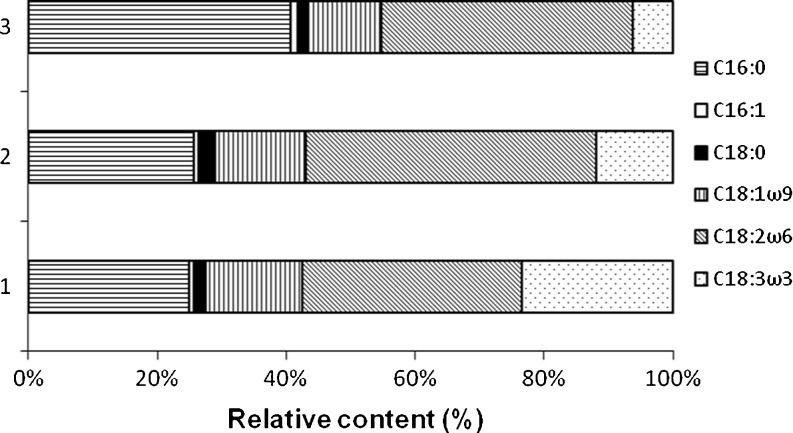
Spectrum of fatty acids palmitic (C16:0), palmitoleic (C16:1), stearic (C18:0), oleic (C18:1ω9), linoleic (C18:2ω6) and α-linolenic (C18:3ω3) given as relative amounts to 100% in *Chlorella* sp. 800 under different cultivation temperatures: 15°C (*1*), 25°C (*2*), 35°C (*3*)

To increase the suitability of a single microalgae strain for biodiesel production, varied cultivation temperature and light intensity were tested, with both high fatty acid productivity and a good quality of biodiesel being equally necessary. A chain length of fatty acids between 16 and 18 C atoms and a low content of unsaturated fatty acids are optimal. At varied cultivation temperature, the spectrum of synthesised fatty acids changed significantly: If growth temperature for *Chlorella* sp. 800 was lowered from 35 to 15°C increased relative content compared to total fatty acids of threefold unsaturated α-linolenic acid (C18:3ω3) from 6.2 to 23.4% was found (Fig. 3). The content of linoleic acid (C18:2ω6) was decreased in the same manner from 39.1 to 34.0% and of palmitic acid (C16:0) from 40.7 to 24.9%. The content of other fatty acids was nearly constant (Fig. 3).

**Fig. 4:**
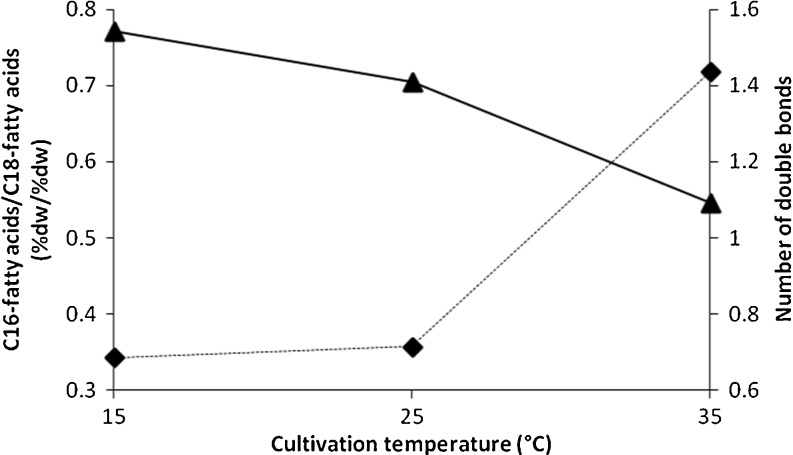
Effect of cultivation temperature on the number of double bonds per fatty acid molecule (*solid line*) and of quotient of summarised C16 fatty acids and summarised C18 fatty acids (*dashed line*) in strain *Chlorella* sp. 800

Summarising these effects in *Chlorella* sp. 800, desaturation of fatty acids was low at a high cultivation temperature. A decrease of the average number of double bonds per fatty acid molecule (1.54 at 15°C, 1.41 at 25°C, 1.09 at 35°C, Fig. 4) was postulated. The chain length of fatty acids was lower at each increase in cultivation temperature. The quotient of summarised C16 fatty acids and summarised C18 fatty acids increased from 0.34 at 15°C to 0.36 at 25°C and to 0.72 at 35°C, mainly a result of increasing content of palmitic acid (Fig. 4). Despite the effect of cultivation temperature, no influence of light intensity on the number of double bonds per fatty acid molecule was detectable (Online Resource 2). Increasing light intensity tended to a slight shortening of the chain length of fatty acids (Online Resource 2). Alongside these effects, the relative amount of fatty acids was largely unchanged (Online Resource 3).

### Productivity of amino acids

**Fig. 5:**
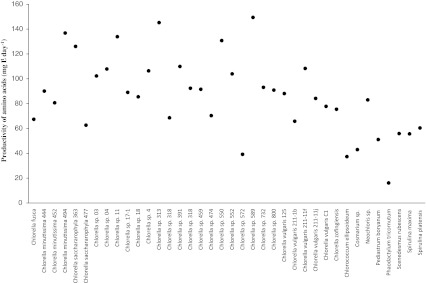
Specific amino acid productivity of 38 screened microalgae strains. For reference strain *Chlorella vulgaris* 132, the productivity of amino acids ranged between 155 and 159 mg L^−1^ day^−1^ over the whole test period

As a result of screening experiments, four strains with an amino acids content of more than 40% dw were identified (Online Resource 1). The blue-green alga *S. platensis* reached a protein content of 46.8% dw and *S. maxima* of 44.9% dw. For green algae *Chlorella* sp. 589, a protein content of 44.3% dw and for *C. saccharophila* 363 a protein content of 42.4% dw were found. Protein content of the reference strain reached values of between 35.7% and 41.9% dw. The main fraction of amino acids consisted of alanine, arginine, aspartic acid, glutamic acid, lysine and leucine. Relative amounts of single amino acids were nearly similar in all screened microalgae strains. The spectrum of main fraction amino acids in the five most productive strains is shown in Online Resource 4. The results indicate that no differences, or only minimal differences, exist in anabolic pathways for amino acids. Results for productivity of amino acids of all screened strains are given in Fig. 5. Outstanding productivity was reached by the reference strain (155–159 mg L^−1^ day^−1^). The next most productive strains were *Chlorella* sp. 589 (149 mg L^−1^ day^−1^), *Chlorella* sp. 313 (145 mg L^−1^ day^−1^) and *C. minutissima* 494 (137 mg L^−1^ day^−1^).

## Discussion

Growth data in this paper concur with already published data: Rodolfi et al. (2008) screened 30 microalgae strains and obtained biomass productivity between 0.04 and 0.37 g L^−1^ day^−1^. Compared to biomass productivity in this study, ranging from 0.06 g L^−1^ day^−1^ (*P. tricornutum*) to 0.495 g L^−1^ day^−1^ (*Chlorella* sp. 800), a good accordance is given. For top biomass producers *Chlorella* sp. and *C. minutissima*, a high practicable suitability for biodiesel production was postulated in literature (Rasoul-Amini et al. 2011; Tang et al. 2011). In addition, *Chlorella* strains have a high fixation capacity for CO_2_ from flue gas generated by combustion processes (Doucha et al. 2005). For *C. minutissima*, a high pollution tolerance has been reported (Bhatnagar et al. 2010) indicating a plausible role in wastewater remediation. The usage of wastewater and industrial fumes for cultivation processes will clearly reduce the production costs of biodiesel production.

In order to test if high biomass productivity excludes high amounts of lipids, fatty acids or amino acids, corresponding correlation analyses for all screened strains were calculated, with no relationship between these data detected (Online Resource 5). The top biomass producer strain *Chlorella* sp. 800, for example, also reached the highest content of fatty acids as well as the third highest lipid content. The *r* ^2^ for all tested strains, all tested in comparison to biomass productivity, was 0.08 for lipids, 0.00 for fatty acids and 0.05 for amino acids. In literature, sometimes a strong negative correlation between lipid content and biomass productivity has been postulated based on the high metabolic cost of lipid biosynthesis (Rodolfi et al. 2008). However, based on screening results of this study, no such effect was detectable.

### Strains with highest lipid productivity

Absolute lipid content of screened microalgae strains ranged from 8% dw (*Pediastrum boryanum*) to 30% dw (*Chlorella* sp. 589) with an average lipid content of 17% dw. In a review paper, Griffiths and Harrison (2009) reported screened microalgae strains with a lipid content of between 5% and 55% dw with an average lipid content of 23% dw. This higher average content was reached due to nine strains (of the 55 tested) containing extremely high lipid contents of more than 30% dw. In this study, there was no possibility of testing these lipid-rich strains. Lipid productivity, of all tested strains, ranged from 10 mg L^−1^ day^−1^ (*P. tricornutum*) to 121 mg L^−1^ day^−1^ (*Chlorella* sp. 800). Studies of existing literature show comparable lipid productivities. Huerlimann et al. (2010) reported lipid productivities between 2 and 23 mg L^−1^ day^−1^ in tested microalgae strains and Rodolfi et al. (2008) between 17 and 61 mg L^−1^ day^−1^. The results were obtained under a light intensity of 100 μmol photons m^−2^ s^−1^ without optimisation of cultivation parameters. Data in this study were generated by a doubled light intensity of 200 μmol photons m^−2^ s^−1^. The resulting higher biomass productivity led to higher lipid productivity.

**Fig. 6:**
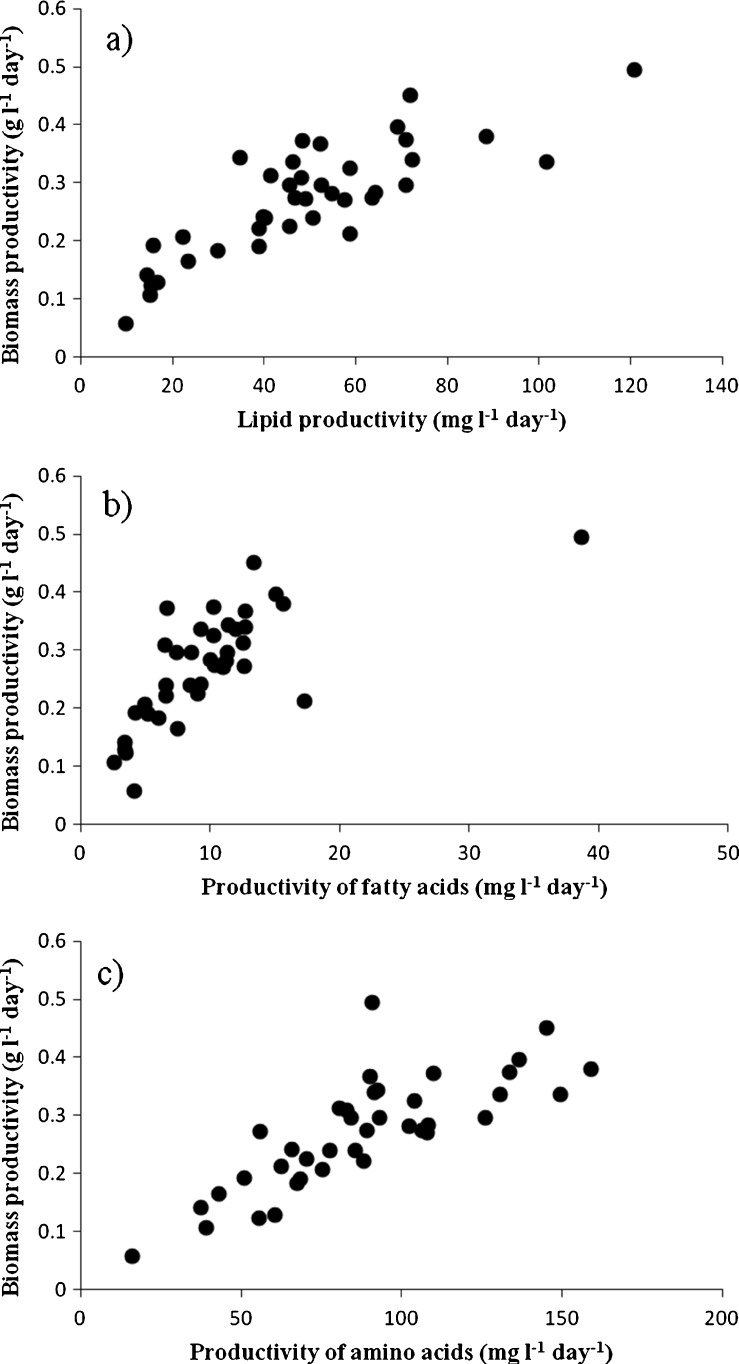
Correlation of biomass productivity with lipid productivity (a; *r* ^2^ = 0.683), with fatty acid productivity (b; *r* ^2^ = 0.510) and with amino acid productivity (c; *r* ^2^ = 0.638) in 39 microalgae strains including the reference strain

The productivity of lipids is the product of biomass productivity and content of lipids. To determine the impact of both values on resulting lipid productivity, correlation studies were undertaken. Biomass productivity influenced the lipid productivity (*r* ^2^ = 0.683; Fig. 6a) more than the lipid content (*r* ^2^ = 0.563; Online Resource 6a), but the distinction was low.

### Most promising strains in terms of productivity of fatty acids

In tested strains, the content of fatty acids ranged from 1.8% dw (*Chlorella* sp. 391*)* to 8.1% dw (*C. saccharophila* 477). Renaud et al. (1994) obtained comparable results for total fatty acid content of microalgae strains ranging from 1.8% to 10.5% dw. For resulting productivity of fatty acids, values between 2.6 mg L^−1^ day^−1^ (*Chlorella* sp. 572) and 38.7 mg L^−1^ day^−1^ (*Chlorella* sp. 800) were calculated. Comparable fatty acid productivity was reported in literature (Lee et al. 2010; Meireles et al. 2008). Please note that these results were achieved under defined non-limiting cultivation conditions. In response to nitrogen deficiency, an accumulation of triacylglycerols containing fatty acids was observed in numerous strains (Basova 2005; Cobelas and Lechado 1989; Merzlyak et al. 2007). It is also worth pointing out that phosphorous limitation or sulphur limitation can lead to increasing amount of fatty acids in algae biomass (Khozin-Goldberg and Cohen 2006; Sato et al. 2000). To estimate the influence of nutrient limitation on screening strains of the current study, further experiments will be conducted.

The correlation investigations gave results as follows: Similar to lipid productivity, the productivity of fatty acids was more influenced by biomass productivity (*r* ^2^ = 0.51; Fig. 6b) than by content of fatty acids (*r* ^2^ = 0.417; Online Resource 6b). The results indicate that biomass productivity is a good indicator of suitability for biodiesel production, but the content of fatty acids is also a factor. For direct transesterification reaction, the amount of fatty acids is crucial. To determine the indication function of lipid content, the correlation between content of fatty acids and content of lipids was compared. A coefficient of determination of *r* ^2^ = 0.29 was estimated (Online Resource 7), with both values only moderately linked with each other. A good accordance was given for *C. saccharophila* 477 (27.6% dw lipids and 8.1% dw fatty acids). In contrast, *Chlorella* sp. 589 reached, with 30.2% dw, the highest lipid content of all screened strains. However, with 3.6% dw, a low content of fatty acids was also recorded. In all tested strains, the fatty acid content was, on average, 20.9 ± 6.4% of lipid content. However, reasons for discrepancy between lipid content and fatty acid content are complex. Based on extraction method, a wide range of lipophilic substances (soluble in chloroform/methanol mixture) were detectable: neutral or polar lipids, wax esters, sterols, hydrocarbons and prenyl derivatives (Hu et al. 2008). In the lipophilic fraction of *Selenastrum gracile*, chlorophyll *a* and *b*, β-carotene, lutein, violaxanthin and neoxanthin were observed (Czygan 1970). Which substances were responsible for high lipid content of *Chlorella* sp. 589 will be determined in further studies.

All identified fatty acids had predominantly even-numbered carbon fatty acid chain lengths, existing as saturated, mono-, di- and tri-unsaturated ones. In accordance for microalgae, most commonly synthesised fatty acids with chain length between C16 and C18 were reported (Hu et al. 2008; Ohlrogge and Browse 1995). In Chlorophyceae, Borowitzka (1997) identified palmitic acid and oleic acid as the main components of fatty acid fraction. Under defined screening conditions, algae mainly synthesise membrane lipids such as glycosylglycerides and phosphoglycerides (Hu et al. 2008; Piorreck and Pohl 1984). Palmitic and oleic fatty acids function as precursors for major constituents of membrane glycerolipids, provided by aerobic desaturation and chain elongation (Erwin 1973). Compared to analysis of *Neochloris oleoabundans* (Tomabene et al. 1983), in nearly all screened strains in the present study, substantial quantities of fatty acids with odd-numbered chain lengths of pentadecanoic acid (C15:0) and of margaric acid (C17:0) were detected. A significant amount of elaidic acid (C18:1 t), a trans-configured fatty acid, was identified in *Chlorella fusca*, *Chlorella* sp. 17-1, *Chlorella* sp. 313, *Chlorella* sp. 418, *C. vulgaris* C1 and *Scenedesmus rubescens*.

With different temperatures of cultivation, different numbers of double bonds in fatty acids were found (Fig. 4). For *Chlorella* sp. 800, the number of double bonds per fatty acid molecule decreased with increasing cultivation temperature. This effect is in accordance to literature data. Sato et al. (1979) described in *Anacystis nidulans* similar changes in the desaturation grade of fatty acids. This is a common strategy in many organisms in an attempt to tolerate various temperatures. By increasing the number of double bounds, the temperature for transition of fluid-to-solid phase of membranes was lowered. In this manner, organisms can prevent adverse solid status. In existing literature, it has been described that the average chain length of fatty acids decreased with a decreasing of temperature. However, in this study and with strain *Chlorella* sp. 800 the opposite was found to be true. The content of C16 fatty acids compared to C18 fatty acids increased with increasing cultivation temperatures (Fig. 4).

The composition of fatty acids strongly influences the quality of biodiesel (Yoo et al. 2010). The most important properties are cetane number, cold-flow properties, oxidative stability and iodine value, derived from the structure of fatty acid methyl esters (Damiani et al. 2010). Despite the high content of polyunsaturated fatty acids in most algal oils (Damiani et al. 2010), fatty acid spectra found in present study are very suitable for use as biodiesel. High amounts of C16 and C18 fatty acids and a high saturation grade were found in the strain with an outstanding productivity for fatty acids *Chlorella* sp. 800 as well as other top producers. Increasing the cultivation temperature indicated an additional quality-increasing effect in the phase of increased lipid synthesis, for instance during nitrogen starvation. A positive influence of increasing light intensity on fatty acid productivity without a quality-downgrading effect on the fatty acid spectrum was furthermore postulated.

### Role of proteins and amino acid spectrum

For economic biodiesel production, residual algae biomass plays an important role. Lipidless biomass contains proteins, which can enhance the amount of nutritions of conventional food preparations (Spolaore et al. 2006). These were analysed in this study.

Contents of amino acids in screened strains ranged from between 18% dw (*Chlorella* sp. 800) and 47% dw (*S. platensis*). Quantification of amino acids described in ‘Materials and methods’ excluded analysis of any tryptophane amount in probes. Despite this fact, contents of summarised amino acids are in accordance with protein contents in literature. Boyd (1973) reported protein contents between 11 and 46% dw in species of fresh water algae. In another publication, the biomass of microalgae contained proteins between 20 and 67% dw (Renaud et al. 1994). These data tend to higher values compared to values reported in this study. A similar lowering of protein contents has been observed by Brown (1991): Continuous illumination conditions decreased protein content. Higher protein contents were obtained under a 12:12 h light–dark cycle. However, proteins are major organic constituents in algae biomass (Online Resource 1) and are the main reason for suitability of food preparations (Cornet 1998; Soletto et al. 2005).

In the screened microalgae strains, all detectable amino acids were quantified. *C. vulgaris* 132, which reached the highest productivity of amino acids, also reached the highest content of all amino acids, ranging from 0.22% dw (cysteine) to 4.5% dw (arginine). For tested *Chlorella* and *Nannochloropsis*, similar amino acid spectra were reported in literature (Sato et al. 1979).

Biomass productivity was clearly identified as the main influencing factor for productivity of amino acids, resulting in high correlation (*r* ^2^ = 0.638; Fig. 6c). For absolute content of amino acids, only a weak correlation of *r* ^2^ = 0.137 was analysed (Online Resource 6c). For increasing productivity of amino acids, the cultivation temperature was varied for *C. vulgaris* 132. Based on high biomass productivity at an optimal temperature of 25°C, the productivity reached 163 mg L^−1^ day^−1^ at its highest value. Productivity decreased at 35°C to 147 mg L^−1^ day^−1^ and at 15°C to 62 mg L^−1^ day^−1^. An influence of cultivation temperature on amino acid spectra was not detected. The relative amount of all amino acids was unchanged during cultivation between 15 and 35°C (Online Resource 8).

## Electronic supplementary material

Below is the link to the electronic supplementary material.

ESM 1 (PDF 145 kb)

## References

[CR1] Basova MM (2005). Fatty acid composition of lipids in microalgae. Int J Algae.

[CR2] Bhatnagar A, Bhatnagar M, Chinnasamy S, Das K (2010). *Chlorella minutissima*—a promising fuel alga for cultivation in municipal wastewaters. Appl Biochem Biotechnol.

[CR3] Borowitzka MA (1997). Microalgae for aquaculture: opportunities and constraints. J Appl Phycol.

[CR4] Boyd CE (1973). Amino acid composition of freshwater algae. Arch Hydrobiol.

[CR5] Brown MR (1991). The amino acid and sugar composition of sixteen species of microalgae used in mariculture. J Exp Mar Biol Ecol.

[CR6] Brown LM, Zeiler BG (1993). Aquatic biomass and carbon dioxide trapping. Energy Convers Manag.

[CR7] Chinnasamy S, Bhatnagar A, Hunt RW, Das KC (2010). Microalgae cultivation in a wastewater dominated by carpet mill effluents for biofuel applications. Bioresour Technol.

[CR8] Chisti Y (2007). Biodiesel from microalgae. Biotechnol Adv.

[CR9] Cobelas MA, Lechado JZ (1989). Lipids in microalgae. A review. I. Biochemistry. Grasas y Aceites.

[CR10] Cornet JF (1998). Le technoscope: les photobioréacteurs. Biofutur.

[CR11] Crutzen PJ, Moiser AR, Smith KA, Winiwarter W (2007). N_2_O release from agro-biofuel production negates global warming reduction by replacing fossil fuels. Atmos Chem Phys Discuss.

[CR12] Czygan FC (1970). Untersuchungen über die Bedeutung der Biosynthese von Sekundär-Carotinoiden als Artmerkmal bei Grünalgen. Arch Mikrobiol.

[CR13] Damiani MC, Popovich CA, Constenla D, Leonardi PI (2010). Lipid analysis in *Haematococcus pluvialis* to assess its potential use as a biodiesel feedstock. Bioresour Technol.

[CR14] Doucha J, Straka F, Lívanský K (2005). Utilization of flue gas for cultivation of microalgae (*Chlorella* sp.) in an outdoor open thin-layer photobioreactor. J Appl Phycol.

[CR15] Erwin JA, Erwin JA (1973). Comparative biochemistry of fatty acids in eukaryotic microorganisms. Lipids and biomembranes of eukaryotic microorganisms.

[CR16] Griffiths MJ, Harrison STL (2009). Lipid productivity as a key characteristic for choosing algal species for biodiesel production. J Appl Phycol.

[CR17] Hammouda O, Gaber A, Abdel-Raouf N (1995). Microalgae and wastewater treatment. Ecotoxicol Environ Saf.

[CR18] Hankamer B, Lehr F, Rupprecht J, Mussgnug JH, Posten C, Kruse O (2007). Photosynthetic biomass and H_2_ production: from bioengineering to bioreactor scale up. Physiol Plantarum.

[CR19] Hoffart MI, Caldeira K, Benford G, Criswell DR, Green C, Herzog H, Jain AK, Kheshgi HS, Lackner KS, Lewis JS, Lightfoot HD, Manheimer W, Mankins JC, Mauel ME, Perkins LJ, Schlesinger ME, Volk T, Wigley TML (2002). Advanced technology paths to global climate stability: energy for a greenhouse planet. Science.

[CR20] Hu Q, Sommerfeld M, Jarvis E, Ghirardi M, Posewitz M, Seibert M, Darzins A (2008). Microalgal triacylglycerols as feedstocks for biofuel production: perspectives and advances. Plant J.

[CR21] Huerlimann R, de Nys R, Heimann K (2010). Growth, lipid content, productivity, and fatty acid composition of tropical microalgae for scale-up production. Biotechnol Bioeng.

[CR22] Khozin-Goldberg I, Cohen Z (2006). The effect of phosphate starvation on the lipid and fatty acid composition of the fresh water eustigmatophyte *Monodus subterraneus*. Phytochemistry.

[CR23] Koonin SE (2006). Getting serious about biofuels. Science.

[CR24] Kruse O, Rupprecht J, Mussgnug JR, Dismukes GC, Hankamer B (2005). Photosynthesis: a blueprint for solar energy capture and biohydrogen production technologies. Photochem Photobiol Sci.

[CR25] Lee JY, Yoo C, Jun SY, Ahn CY, Oh HM (2010). Comparison of several methods for effective lipid extraction from microalgae. Bioresour Technol.

[CR26] Mallick N (2002). Biotechnological potential of immobilized algae for wastewater N, P and metal removal: a review. Biometals.

[CR27] Meireles LA, Guedes AC, Barbosa CR, Azevedo JL, Cunha JP, Malcata FX (2008). On-line control of light intensity in a microalgal bioreactor using a novel automatic system. Enz Microb Technol.

[CR28] Merzlyak MN, Chivkunova OB, Gorelova OA, Reshetnikova IV, Solovchenko AE, Khozin-Goldberg I, Cohen Z (2007). Effect of nitrogen starvation on optical properties, pigments, and arachidonic acid content of the unicellular green alga *Parietochloris incisa* (Trebouxiophyceae, Chlorophyta). J Phycol.

[CR29] Muñoz R, Guieysse B (2006). Algal–bacterial processes for the treatment of hazardous contaminants: a review. Water Res.

[CR30] Ohlrogge J, Browse J (1995). Lipid biosynthesis. Plant Cell.

[CR31] Pacala S, Socolow R (2004). Stabilization wedges: solving the climate problem for the next 50 years with current technologies. Science.

[CR32] Patil V (2007). The relevance of biofuels. Curr Sci.

[CR33] Patil V, Tran KQ, Giselrød HR (2008). Towards sustainable production of biofuels from microalgae. Int J Mol Sci.

[CR34] Piorreck M, Pohl P (1984). Formation of biomass, total protein, chlorophylls, lipids and fatty acids in green and blue-green algae during one growth phase. Phytochem.

[CR35] Pirt SJ (1986). The thermodynamic efficiency (quantum demand) and dynamics of photosynthetic growth. New Phytol.

[CR36] Pulz O (2001). Photobioreactors: production systems for phototrophic microorganisms. Appl Microbiol Biotechnol.

[CR37] Pulz O, Gross W (2004). Valuable products from biotechnology of microalgae. Appl Microbiol Biotechnol.

[CR38] Rasoul-Amini S, Montazeri-Najafabady N, Mobasher MA, Hoseini-Alhashemi S, Ghasemi Y (2011) *Chlorella* sp.: a new strain with highly saturated fatty acids for biodiesel production in bubble-column photobioreactor. Appl Energy. doi:10.1016/j.apenergy.2010.12.040

[CR39] Renaud SM, Parry DL, Thinh LV (1994). Microalgae for use in tropical aquaculture I: gross chemical and fatty acid composition of twelve species of microalgae from the Northern Territory, Australia. J Appl Phycol.

[CR40] Richmond A (2000). Microalgal biotechnology at the turn of the millennium: a personal view. J Appl Phycol.

[CR41] Righelato R, Spracklen DV (2007). Carbon mitigation by biofuels or by saving and restoring forests?. Science.

[CR42] Rodolfi L, Zittelli GC, Bassi N, Padovani G, Biondi N, Bonini G, Tredici MR (2008). Microalgae for oil: strain selection, induction of lipid synthesis and outdoor mass cultivation in a low-cost photobioreactor. Biotechnol Bioeng.

[CR43] Sato N, Murta N, Miura Y, Ueta N (1979). Effect of growth temperature on lipid and fatty acid compositions in the blue-green algae *Anabaena variabilis* and *Anacystis nidulans*. Biochim Biophys Acta.

[CR44] Sato N, Hagio M, Wada H, Tsuzuki M (2000) Environmental effects on acidic lipids of thylakoid membranes. In: Harwood JL and Quinn, PJ (eds) Recent advances in the biochemistry of plant lipids. Portland, London, pp 912–914 11171255

[CR45] Schaub G, Kolb T, Steiger W (2007). Biofuels. Second-generation biofuels. Chemie Ingenieurtechnik.

[CR46] Schenk PM, Thomas-Hall SR, Stephens E, Marx UC, Mussgnug JH, Posten C, Kruse O, Hankamer B (2008). Second generation biofuels: high-efficiency microalgae for biodiesel production. Bioenerg Res.

[CR47] Shay EG (1993). Diesel fuel from vegetable oils: status and opportunities. Biomass Bioenergy.

[CR48] Soletto D, Binaghi L, Lodi A, Carvalho JCM, Converti A (2005). Batch and fed-batch cultivations of *Spirulina platensis* using ammonium sulphate and urea as nitrogen sources. Aquaculture.

[CR49] Spolaore P, Joannis-Cassan C, Duran E, Isambert A (2006). Commercial applications of microalgae. J Biosci Bioeng.

[CR50] Tang H, Chen M, Garcia MED, Abunasser N, Ng KYS, Salley SO (2011). Culture of microalgae *Chlorella minutissima* for biodiesel feedstock production. Biotechnol Bioeng.

[CR51] Tomabene TG, Holzer G, Lien S, Burris N (1983). Lipid composition of the nitrogen starved green alga *Neochloris oleoabundans*. Enzyme Microb Technol.

[CR52] Ugwu CU, Aoyagi H, Uchiyama H (2008). Photobioreactors for mass cultivation of algae. Bioresour Technol.

[CR53] Van Gerpen J, Mielenze JR (2009). Biofuel. Methods in molecular biology. Biodiesel: small scale production and quality requirements.

[CR54] Xu L, Weathers PJ, Xiong XR, Liu CZ (2009). Microalgal bioreactors: challenges and opportunities. Eng Life Sci.

[CR55] Yoo C, Jun SY, Lee JY, Ahn CY, Oh HM (2010). Selection of microalgae for lipid production under high levels carbon dioxide. Bioresour Technol.

