# Supplementary material for: Biomass productivity and productivity of fatty acids and amino acids of microalgae strains as key characteristics of suitability for biodiesel production
Source: J Appl Phycol. 2012 Feb 11;24(6):1407–18. doi: 10.1007/s10811-012-9795-3 (PMC3478515; doi:10.1007/s10811-012-9795-3)
Supplement: Supplementary file 1 — (PDF 145 kb) [file 10811_2012_9795_MOESM1_ESM.pdf]

Online Resources

Online Resource 1 Content of lipids (filled circles), fatty acids (open circles) and amino acids (open triangles) of 38 screened microalgae strains. For reference strain *Chlorella vulgaris* 132 the content of lipids ranged between 18.5 and 23.3% dw, of fatty acids between 3.1 and 4.1% dw and of amino acids between 35.7 and 41.9% dw over the whole test period

Online Resource 2 Effect of light intensity on the number of double bonds per fatty acid molecule (*solid line*) and on the quotient of summarised C16-fatty acids and summarised C18-fatty acids (*dashed line*) in strain *Chlorella sp.* 800

Online Resource 3 Spectrum of fatty acids palmitic (C16:0), palmitoleic (C16:1), stearic (C18:0), oleic (C18:1ω9), linoleic (C18:2ω6), α-linolenic (C18:3ω3) given as relative amounts to 100% in *Chlorella sp.* 800 under different light intensities: 200 µmol photons m^{-2} s^{-1} (*1*), 300 µmol photons m^{-2} s^{-1} (*2*), 400 µmol photons m^{-2} s^{-1} (*3*), 500 µmol photons m^{-2} s^{-1} (*4*)

Online Resource 4 Spectrum of amino acids alanine, arginine, aspartic acid, glutamic acid, lysine, leucine given as relative amounts to 100% in five microalgal strains with highest amino acid productivity. *Chlorella vulgaris* 132 (*1*), *Chlorella sp.* 589 (*2*), *Chlorella sp.* 313 (*3*), *Chlorella minutissima* 494 (*4*), *Chlorella sp.* 11 (*5*)

Online Resource 5 Correlation of biomass productivity with lipid content (a; r^2=0.08), with content of fatty acids (b; r^2=0.00) and with content of amino acids (c; r^2=0.05) in 39 microalgae strains including the reference strain

Online Resource 6 Correlation of productivity of lipids with content of lipids (a; r^2=0.56), of productivity of fatty acids with content of fatty acids (b; r^2=0.42) and of productivity of amino acids with content of amino acids (c; r^2=0.14) in 39 microalgae strains including the reference strain

Online Resource 7 Correlation of lipid content with content of fatty acids (r^2=0.29) in 39 microalgae strains including the reference strain

Online Resource 8 Spectrum of amino acids alanine, arginine, aspartic acid, glutamic acid, lysine, leucine given as relative amounts to 100% in *Chlorella sp.* 800 under different cultivation temperatures: 15°C (*1*), 25°C (*2*), 35°C (*3*)

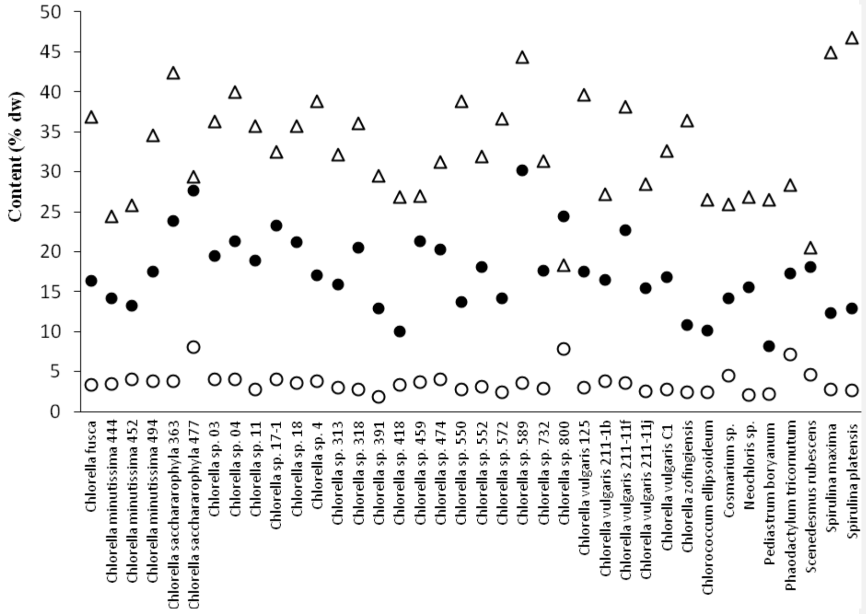

Online Resource 1 Content of lipids (filled circles), fatty acids (open circles) and amino acids (open triangles) of 38 screened microalgae strains. For reference strain *Chlorella vulgaris* 132 the content of lipids ranged between 18.5 and 23.3% dw, of fatty acids between 3.1 and 4.1% dw and of amino acids between 35.7 and 41.9% dw over the whole test period

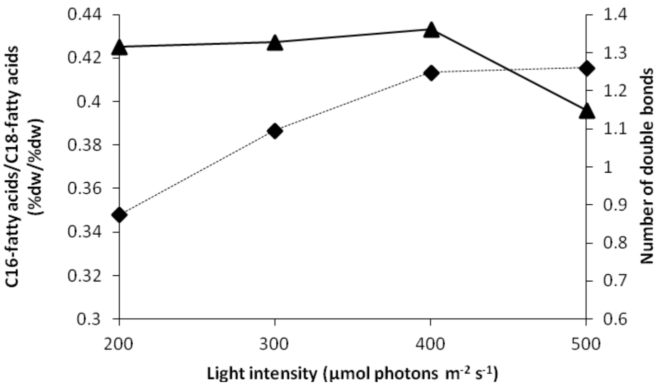

Online Resource 2 Effect of light intensity on the number of double bonds per fatty acid molecule (*solid line*) and on the quotient of summarised C16-fatty acids and summarised C18-fatty acids (*dashed line*) in strain *Chlorella sp.* 800

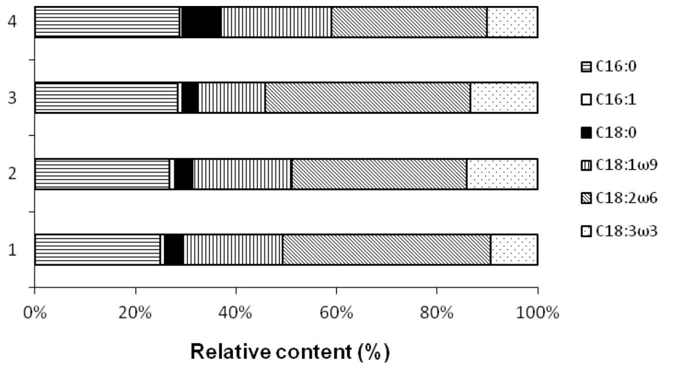

Online Resource 3 Spectrum of fatty acids palmitic (C16:0), palmitoleic (C16:1), stearic (C18:0), oleic (C18:1ω9), linoleic (C18:2ω6), α-linolenic (C18:3ω3) given as relative amounts to 100% in *Chlorella sp.* 800 under different light intensities: 200 µmol photons m^{-2} s^{-1} (*1*), 300 µmol photons m^{-2} s^{-1} (*2*), 400 µmol photons m^{-2} s^{-1} (*3*), 500 µmol photons m^{-2} s^{-1} (*4*)

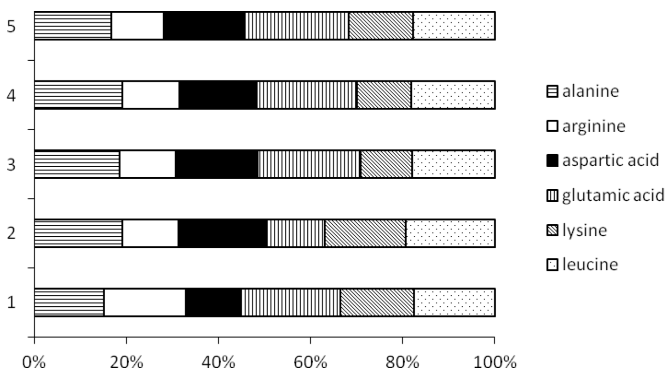

Online Resource 4 Spectrum of amino acids alanine, arginine, aspartic acid, glutamic acid, lysine, leucine given as relative amounts to 100% in five microalgal strains with highest amino acid productivity. *Chlorella vulgaris* 132 (*1*), *Chlorella sp.* 589 (*2*), *Chlorella sp.* 313 (*3*), *Chlorella minutissima* 494 (*4*), *Chlorella sp.* 11 (*5*)

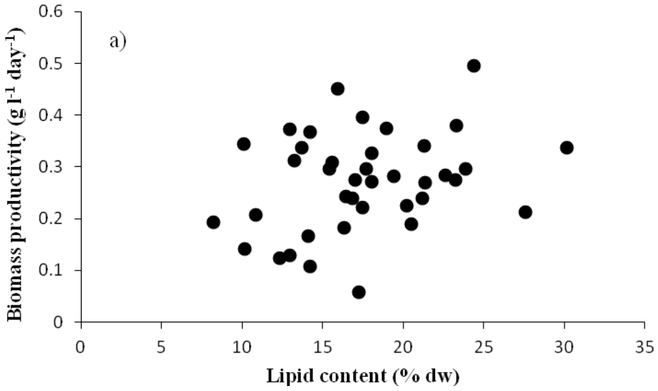

a)
Biomass productivity (g l-1 day-1)
Lipid content (% dw)

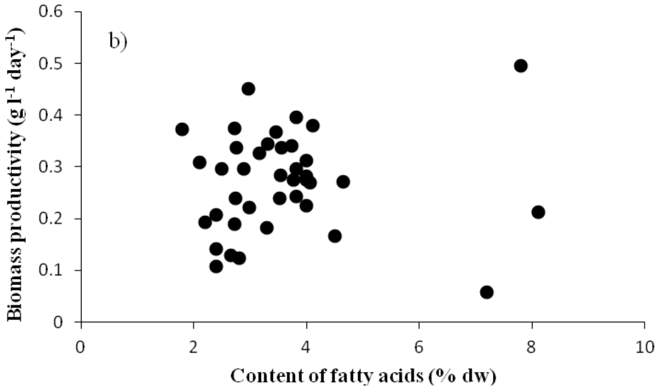

b)
Biomass productivity (g l-1 day-1)
Content of fatty acids (% dw)

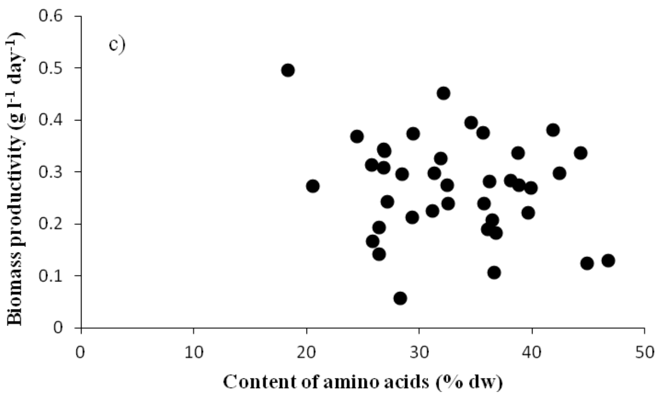

Online Resource 5 Correlation of biomass productivity with lipid content (a; r^2=0.08), with content of fatty acids (b; r^2=0.00) and with content of amino acids (c; r^2=0.05) in 39 microalgae strains including the reference strain

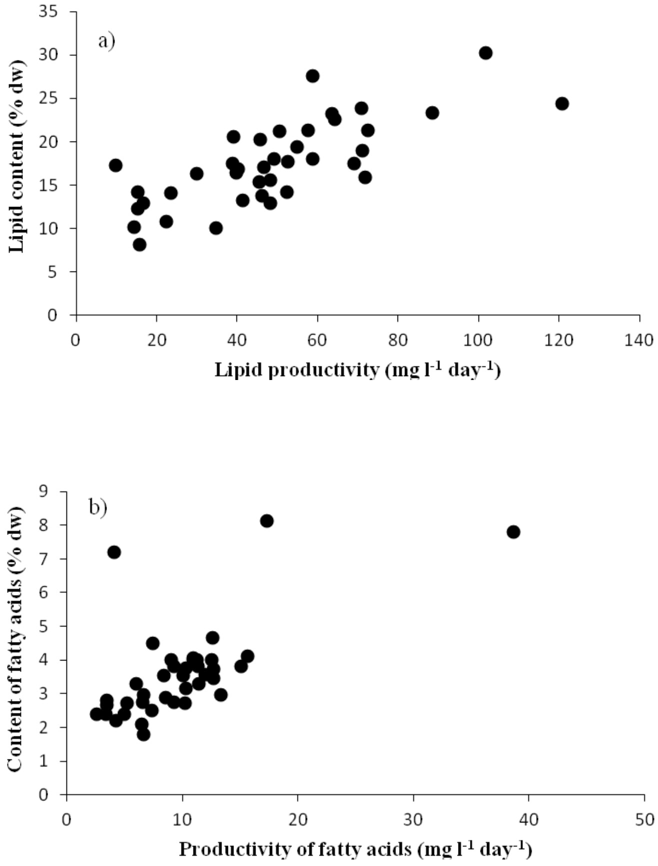
a)
Lipid content (% dw)
Lipid productivity (mg l-1 day-1)
0
5
10
15
20
25
30
35
0
20
40
60
80
100
120
140
b)
Content of fatty acids (% dw)
Productivity of fatty acids (mg l-1 day-1)
0
1
2
3
4
5
6
7
8
9
0
10
20
30
40
50

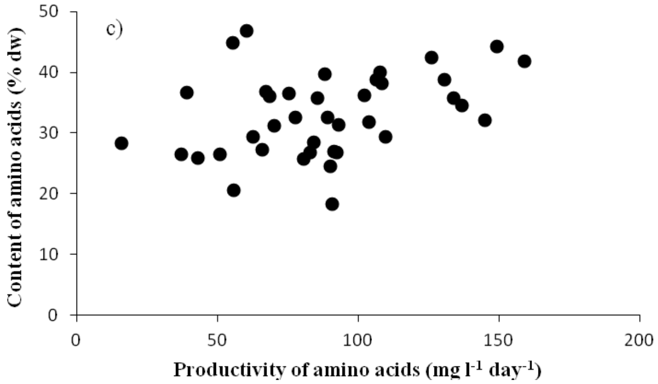

Online Resource 6 Correlation of productivity of lipids with content of lipids (a; r^2=0.56), of productivity of fatty acids with content of fatty acids (b; r^2=0.42) and of productivity of amino acids with content of amino acids (c; r^2=0.14) in 39 microalgae strains including the reference strain

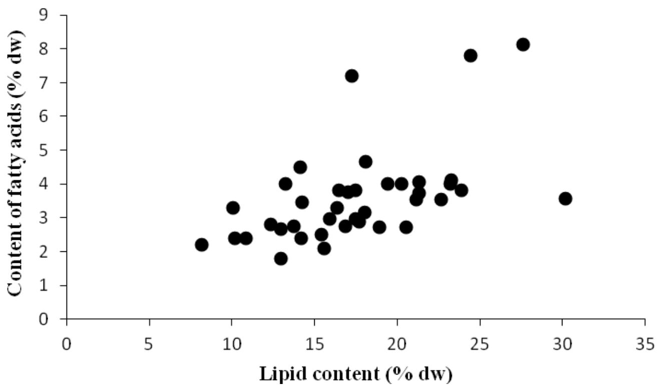

Online Resource 7 Correlation of lipid content with content of fatty acids (r^2=0.29) in 39 microalgae strains including the reference strain

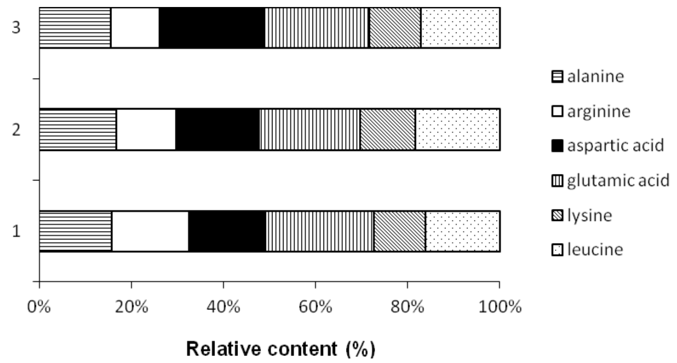

Online Resource 8 Spectrum of amino acids alanine, arginine, aspartic acid, glutamic acid, lysine, leucine given as relative amounts to 100% in *Chlorella sp.* 800 under different cultivation temperatures: 15°C (*1*), 25°C (*2*), 35°C (*3*)
